# Ibuprofen as a corrosion inhibitor for copper in synthetic acid rain solution

**DOI:** 10.1038/s41598-019-51299-2

**Published:** 2019-10-11

**Authors:** Zaklina Z. Tasić, Marija B. Petrović Mihajlović, Ana T. Simonović, Milan B. Radovanović, Milan M. Antonijević

**Affiliations:** University of Belgrade, Technical Faculty in Bor P.O. Box 50, 19210 Bor, Serbia

**Keywords:** Metals and alloys, Environmental impact

## Abstract

It is known that if unused drugs are improperly disposed, they can pollute the environment. Furthermore, researchers are still trying to find an environmentally friendly corrosion inhibitor. These factors lead to the possible application of unused pharmaceutical compounds as corrosion inhibitors. The feasibility of an anti-inflammatory, analgesic and antipyretic drug, ibuprofen, was evaluated as a potential copper corrosion inhibitor in synthetic acid rain solution. This investigation was performed by applying electrochemical and weight loss measurements and quantum chemical calculations. The results obtained by these techniques revealed the ability of ibuprofen to protect copper from corrosion. The inhibition efficiency of ibuprofen rises with increase in its concentration and can reach a value of 97.3%. The results of surface analysis of treated coupons by scanning electron microscopy and theoretical calculations are consistent with the experimental results.

## Introduction

Generally, several metals and alloys such as copper, brass, aluminum, tin and steel are used in various industrial fields. Copper possesses thermal and electrical conductivity so it is used in heat exchangers, and as a conductor in the electronic industry. In addition, copper is also used for coating sculptures and roofs. Regardless of the place of application, copper dissolution can occur in aggressive environments^1–3^. This has negative consequences for the properties of the metal, and can leads to significant economic losses. Based on many studies^4–7^, different corrosion inhibitors have been developed that can be used for the protection of copper. These inhibitors can be inorganic^8,9^ or organic compounds^10,11^. Due to the low inhibition efficiency of inorganic compounds^9^, different classes of organic compounds have been investigated for this purpose among which the most important are azole compounds. In the presence of azole and azole derivatives such as imidazole^12–14^, triazole^15–18^, pyrazole^19,20^, tetrazole^21–23^, thiazole^24–26^ and thiadiazole^10,27,28^, significant reductions in the corrosion of copper have been achieved. However, several of these compounds are toxic to the environment. Therefore, research has been directed towards finding environmentally acceptable corrosion inhibitors. There are numerous studies indicating that amino acids^29–31^, purine and its derivatives^32–37^, plant extracts^38–40^ and pharmaceutical compounds^41–44^ are potentially environmentally friendly corrosion inhibitors for copper in a variety of media such as chloride, nitrate and sulfate solutions.

Researchers are working to find an efficient, low cost and non-toxic corrosion inhibitor. According to the investigations^45,46^, traditional inhibitors could be replaced by pharmaceuticals. By comparing the price of the pharmaceutical active compounds and other organic compounds, active compounds of drugs are more costly. Among drugs, natural compounds like plant extracts are attractive as green corrosion inhibitors^47,48^. In order to obtain a plant extract, various techniques such as extraction are involved which increase the coast while expired pharmaceuticals are available^49,50^. It is worth noting that after the expiration date, higher than 90% of active component of drug remains stable for a long time^51^. Further, an expired drug can be realized in the environment due to inappropriate disposal. Drugs are brought in the environment through household waste or toilet^52–54^. The pharmaceuticals that are unsuitable for further human usage should be degraded by adequate technique. In accordance with these, using of expired drugs as possible corrosion inhibitors can decrease environmental pollution and also reduce the degradation costs.

Various research groups^52–56^ concluded that pharmaceutical industries are also responsible for disposing expired or unused drugs into the environment. Having this in mind, unused drugs should be degraded by using an adequate technique like photochemical process^57,58^, biodegradation process^59,60^, adsorption^61^ and nanofiltration process^62^. According to the obtained results by Kanama^63^, and Paxeus^64^ the carrousel-type activated sludge system decreased the influent concentrations of all target PPCPs by 40–98% before their eventual discharge. Taking into account the numerous investigations^42–44,50^ about using drugs as possible corrosion inhibitors, it may be assumed that these compounds have ability to adsorb on metal surface and form complexes with metals. On that way, the reuse of drugs limits the environmental pollution and reduces the disposal costs, so they have prompted interest for use in corrosion testing.

Ibuprofen (2-(p-isobuthylphenyl) propionic acid) is an anti-inflammatory, analgesic and antipyretic drug largely used in the treatment of muscle and head pain, inflammation in rheumatic disease and for the treatment of fever^65^. With this in mind, it is interesting to examine the ability of expired ibuprofen to protect copper from corrosion in synthetic acid rain solution.

## Materials and Methods

### Electrochemical measurements

Electrochemical measurements were performed using potentiostat (IVIUM XRE, IVIUM Technologies) with the appropriate software in a three electrode configuration. Copper electrode with exposed surface area of 0.49 cm^2^ was used as the working electrode, while a standard calomel electrode (SCE) and a platinum wire were used as the reference and auxiliary electrodes, respectively. Prior to each measurement, the copper electrode was polished with alumina (0.3μm Al_2_O_3_, Buehler USA), washed with distilled water and then dried.

The following electrochemical methods were used in the research: open circuit potential (OCP) measurements, potentiodynamic polarization, cyclic voltammetry, electrochemical impedance spectroscopy and beside these weight loss measurements. The open circuit potential was measured for 30 minutes and before the potentiodynamic polarization measurements were performed. The polarization measurement was recorded in the anodic direction from the open circuit potential to 0.25 V (vs. SCE) as well as in the cathodic direction from the open circuit potential to −0.25 V (vs. SCE). Cyclic voltammetry was performed over potential range of −1 V (vs. SCE) to 1 V (vs. SCE). The scan rate was 1 mV/s for the potentiodynamic polarization measurements and 10 mV/s for the cyclic voltammetry measurements. Electrochemical impedance spectroscopy measurements were conducted at open circuit potential over a frequency range of 10 kHz – 0.01 Hz, with a single amplitude perturbation of 10 mV using IVIUM soft.

The synthetic acid rain solution (SAR) was prepared using the following compounds: Na_2_SO_4_ (0.2 g/l) (Zorka Pharmacy, Serbia), NaHCO_3_ (0.2 g/l) (Zorka Pharmacy, Serbia) and NaNO_3_ (0.2 g/l) (Zorka Pharmacy, Serbia)^66^. A pH value of 2.42 for the synthetic acid rain was achieved by the addition of an H_2_SO_4_ solution. Inhibitor solutions of ibuprofen were prepared by dissolving the required amount of ibuprofen in the synthetic acid rain solution in order to obtain concentration of 1·10^–2^M. The solution with the highest concentration was diluted in order to obtain solutions with lower concentrations (5·10^−3^M, 1·10^−3^M and 5·10^−4^M). In our investigation expired ibuprofen syrup is used (purchased at a local pharmacy). Based on the drug specification where the content of the active substance (ibuprofen) is 100 mg in 5 ml of syrup, the calculation for the concentration of 1·10^−2^M was made. Further, the appropriate volume of ibuprofen syrup was dissolved in synthetic acid rain solution.

### Weight loss measurements

Copper specimens 30 × 30 × 0.5 mm in dimension were used in the weight loss experiments. These samples were immersed in synthetic acid rain solutions in the absence and presence of various concentrations of ibuprofen for five days at room temperature. Before immersion, each sample was polished with emery paper, washed with ethanol and distilled water and then weighed (analytical balance OHAUS PA214CM; accuracy of weighing process 0.0001 g). After treatment in the test solutions, the copper samples are withdrawn, washed, dried and then reweighed. The weight loss measurements are triplicated.

### Analysis of copper surfaces by scanning electron microscopy with energy dispersive spectroscopy

The surface characterization of the copper samples treated in different acid solutions was carried out to confirm the protective ability of the ibuprofen. For this purpose a Tescan VEGA 3 LM scanning electron microscope with Oxford EDS X-act Inca 350 system was used. The samples were prepared using the same methods used for the weight loss measurements and after being immersed for five days in the test solutions the surface characterization of the samples was performed.

## Results and Discussion

### Open circuit potential and potentiodynamic polarization measurements

**Figure 1:**
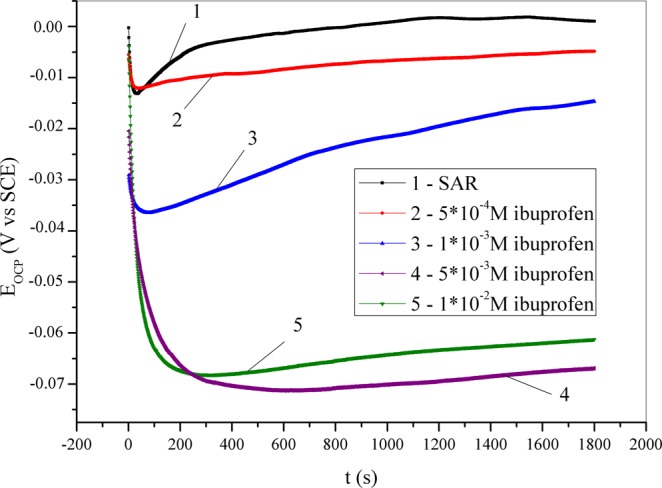
Open circuit potential curves for copper recorded in synthetic acid rain solutions without and with the addition of ibuprofen.

Determination of the open circuit potential (OCP) values for copper in synthetic acid rain solution without and with the addition of an inhibitor was performed for 30 minutes, and obtained curves are shown in Fig. 1. At the beginning of measurement in the blank solution, the OCP is less significantly shifted to more negative values compared with the trends in the presence of ibuprofen. This is the result of the deposition of corrosion products on the copper surface. This shift is more obvious with increase in the inhibitor concentration and could be explained by the adsorption of inhibitors on the active corrosion sites of the copper surface^67^. By comparing the OCP values obtained at the end of the experiments in uninhibited and in inhibited solutions, the ibuprofen could be classified as a mixed-type inhibitor with a more pronounced effect on the cathodic process^68–70^.

**Figure 2:**
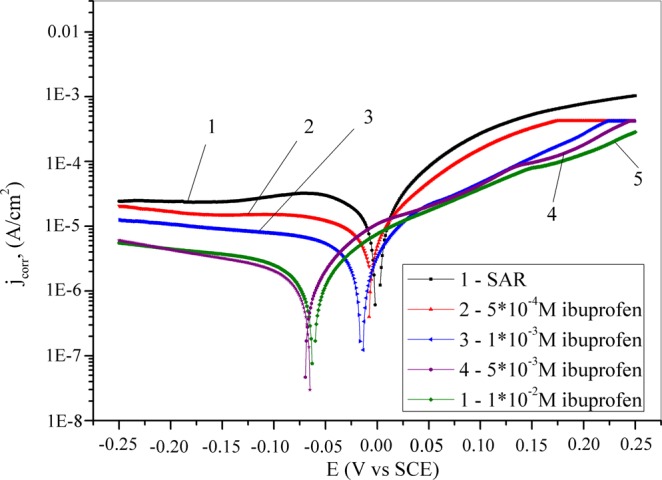
Potentiodynamic polarization curves for copper recorded in synthetic acid rain solution without and with the addition of ibuprofen.

After determining the OCP values, potentiodynamic polarization curves were recorded in both the anodic and cathodic directions. The obtained potentiodynamic polarization curves for copper in synthetic acid rain solutions without and with the addition of inhibitor are shown in Fig. 2. On the basis of the presented curves, it is obvious that the corrosion current density (j_corr_) is reduced in the presence of the expired drug. The corrosion potential (*E*_*corr*_), is shifted in the negative direction in the inhibited solution in comparison to the E_corr_ of the blank solution. This parameter becomes more negative with increase in the inhibitor concentration. However, the change of E_corr_ in inhibited solutions is lower than 85 mV in regard to E_corr_ value in blank solution. Based on the literature^71,72^ if the displacement of E_corr_ in inhibited solution is higher than 85 mV compared to E_corr_ value in uninhibited solution, the tested compound is classified as an anodic or cathodic type. However, if this change in E_corr_ values is less than 85 mV, it is about mixed type. Thus, ibuprofen can be classified as mixed type inhibitor.

A similar conclusion was observed for the open circuit potential measurements. The obtained data for the corrosion potentials as well as the corrosion current densities, anodic (*b*_*a*_) and cathodic (*b*_*c*_) Tafel slopes, polarization resistance (*R*_*p*_) and inhibition efficiencies (*IE*) are presented in Table 1.

**Table 1:** Electrochemical parameters of copper oxidation in SAR solution and with the addition of different concentrations of ibuprofen.

Inhibitor concentration, M	E_corr_, V vs SCE	j_corr_, μA/cm^2^	b_a_, V/dec	-b_c_, V/dec	R_p,_ kΩ·cm^2^	IE, %
/	−0.0014	10.4	0.057	0.102	1.5	/
5·10^−4^	−0.0055	3.76	0.045	0.088	3.5	63.8
1·10^−3^	−0.017	0.868	0.027	0.022	8.8	91.6
5·10^−3^	−0.067	0.392	0.031	0.049	26.2	96.2
1·10^−2^	−0.064	0.287	0.033	0.047	29.3	97.2

The parallel cathodic Tafel lines in SAR containing ibuprofen indicated that the presence of this compound does not modify the cathodic reaction^73^. Additionally, the values of b_a_ and b_c_ changed with the addition of the inhibitor because of the adsorption of inhibitor molecules on the metal surface to form protective layer^74^. By analyzing the polarization resistance in Table 1, it can be said that this parameter increases upon the addition of inhibitors. R_p_ also rises with increased inhibitor concentration. The addition of ibuprofen in SAR leads to decreased current density, which becomes more pronounced with increases in the ibuprofen concentration (Fig. 2). This shows that ibuprofen can to protect copper under these conditions. The calculated values of inhibition efficiency and polarization resistance using Eqs. (1) and (2) confirm the inhibitory properties of the expired ibuprofen^75,76^. The inhibition action is related to the adsorption of inhibitor molecules on the copper surface and is dependents on the inhibitor concentration.

1 $$IE=[({j}_{corr}-{j}_{corr(inh)})/{j}_{corr}]\cdot 100$$

Where *j*_*corr*_ and *j*_*corr(inh)*_ are corrosion current densities in the absence and presence of the inhibitor, respectively.

Polarization resistance values were calculated according to Stern-Geary equation:^77,78^

2 $${R}_{p}=[({b}_{a}\cdot {b}_{c})/(2.303\cdot ({b}_{a}+{b}_{c}))]\cdot 1/{j}_{corr}$$

### Cyclic voltammetry

**Figure 3:**
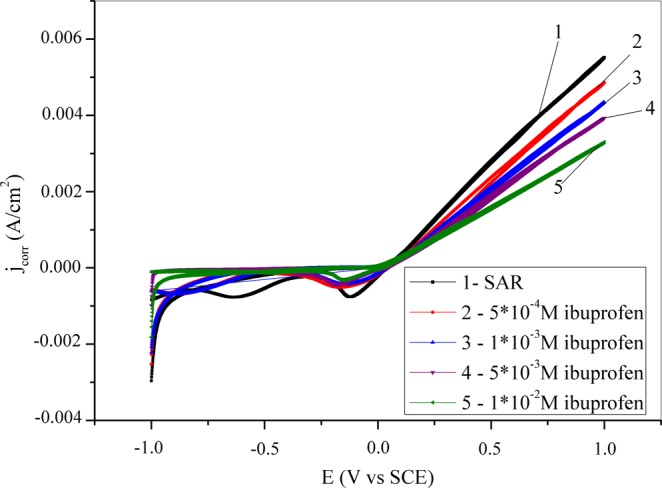
Cyclic voltammetry curves for copper recorder in synthetic acid rain solution without and with the addition of ibuprofen.

Another electrochemical method used to examine the inhibitory ability of ibuprofen in synthetic acid rain is cyclic voltammetry. This method is performed over a wider potential range than potentiodynamic polarization, and obtained curves are shown in Fig. 3. The curves obtained in the inhibitor-free solution indicate the dissolution of copper and the formation of Cu^+^ ions (reaction 3). Furthermore, the current density increases with the potential due to the formation of Cu^2+^ ions (reaction 4)^79^. A similar mechanism of copper dissolution in SAR has been proposed by Magaino^80^. Additionally, formed Cu^+^ ions can react with anionic species (X^n-^) present in the SAR solution by reaction (5). In the reverse scan two cathodic peaks are observed corresponding to the reduction of the formed copper species.

3 $$Cu\to C{u}^{+}+{e}^{-}$$

4 $$C{u}^{+}\to C{u}^{2+}+{e}^{-}$$

5 $$nC{u}^{+}+{X}^{n-}\to {[{(C{u}^{+})}_{n}{X}^{n-}]}_{surf}$$

In the presence of the lowest concentration of inhibitor, the copper surface is not adequately covered so the dissolution continues. However, the addition of higher concentrations of inhibitor (5·10^−3^M and 1·10^−2^M) leads to higher copper surface coverage and the current density is significantly reduced in comparison to the blank solution. Additionally, the decrease of cathodic peak intensity relative to the inhibitor-free solution points to the protective effect of the ibuprofen^81^. Furthermore, the second cathodic peak is not evident in the presence of ibuprofen which indicates the irreversibility of the process.

### Electrochemical impedance spectroscopy

**Figure 4:**
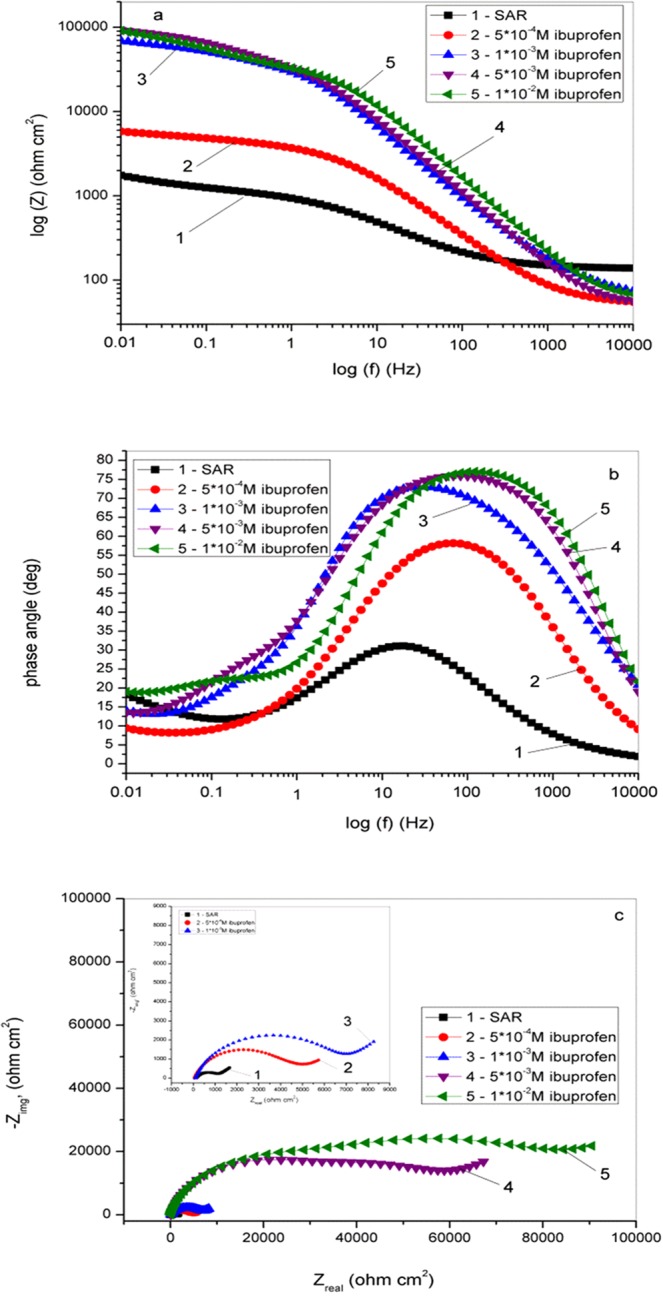
EIS diagrams for copper in SAR in the presence of different concentrations of ibuprofen: (**a**) Bode modules (**b**) Bode phase angle and (**c**) Nyquist plots.

In order to investigate in more detail the influence of ibuprofen on the corrosion behavior of copper in SAR, electrochemical impedance spectroscopy experiments were carried out. The obtained results are shown in Fig. 4(a–c). According to these figures, EIS parameters obtained by fitting are summarized in Table 2. By analyzing the Nyquist diagram (Fig. 4c), it can be seen that semicircle diameter increases as increases the concentration of inhibitor. Thus, the corrosion rate is reduced^82^. Additionally, in the low frequency area, Warburg impedance is observed indicating the diffusion processes, i.e. diffusion of dissolved oxygen or other corrosive species to the surface of copper^83^ or the diffusion of soluble copper species^84^.

**Table 2:** Electrochemical impedance spectroscopy parameters for copper in SAR solution without and with the addition of ibuprofen.

Inhibitor, M	R_s_, Ω cm^2^	R_f_, Ω cm^2^	R_ct_, Ω cm^2^	C_f_, μF cm^−2^	n_1_	C_dl_, μF cm^−2^	n_2_	W, Ω^−1^ cm^−2^ s^0.5^	IE,%	χ^2^
/	125.6	900.9	998.6	0.353	0.70	74.5	0.50	102.4	/	1.0·10^−3^
5·10^−4^	51.54	3278	1893	0.251	0.79	1.15	0.78	236.1	63.2	1.3·10^−3^
1·10^−3^	92.68	19830	27840	0.310	0.83	0.213	0.74	2423	96.0	1.1·10^−3^
5·10^−3^	50.25	39470	29200	0.182	0.89	0.349	0.76	3890	97.2	1.0·10^−3^
1·10^−2^	60.07	31610	39090	0.113	0.89	0.059	0.72	5549	97.3	1.2·10^−3^

In addition to the Nyquist diagram, Bode plots are shown in Fig. 4(a,b). In accordance with these figures, it is obvious that impedance values have increasing trend in the whole frequency area with the addition of ibuprofen. The increasing trend of impedance is related with ibuprofen inhibitory ability^83,84^. Furthermore, Bode phase plots show that phase angle is higher in the presence of inhibitor in comparison to the phase angle in SAR that implies the inhibition of copper dissolution.

**Figure 5:**
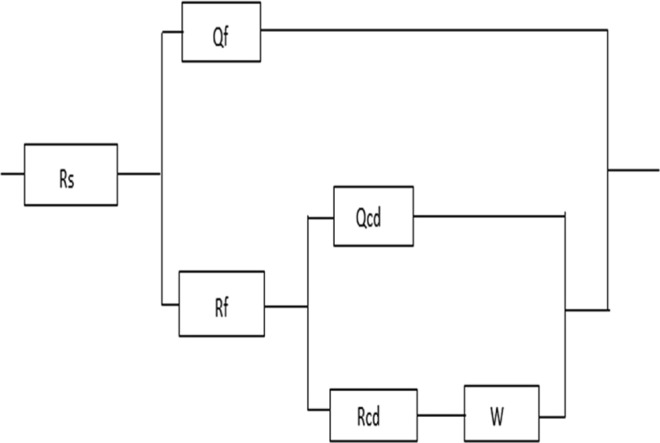
Electrical equivalent circuit for copper in SAR solution in the absence and presence of different concentration of ibuprofen.

The IVIUM soft program and the equivalent circuit shown in Fig. 5 were used for fitting experimental data where R_s_ is the solution resistance, R_f_ is the resistance of protective inhibitor film formed on copper surface, R_ct_ is the charge transfer resistance, Q_f_ and Q_dl_ represent CPE – constant phase elements, C_f_ represents film capacitance and C_dl_ is double layer capacitance, W is the Warburg impedance and n represents deviation parameter^42,85^. C_f_ and C_dl_ parameters are calculated according to the Eqs. (6) and (7):

6 $${C}_{f}={({Q}_{f}{R}_{f}^{1-{n}_{1}})}^{1/{n}_{1}}$$

7 $${C}_{dl}={({Q}_{dl}{R}_{ct}^{1-{n}_{2}})}^{1/{n}_{2}}$$

According to the results shown in Table 2, n values increase in the presence of ibuprofen which indicates the increase of the surface homogeneity due to the adsorption of inhibitor^84^. Furthermore, as the concentration of inhibitor increased, values of C_f_ and C_dl_ decreased, while values of W increased. This is related with the adsorption of inhibitor molecules on the copper surface leading to decrease exposed copper surface to aggressive ions. According to the Iroh and Su^86^ and Ameer *et al*.^87^ and also according to the obtained results, it is assumed that the copper surface is uniformly coated.

Inhibition efficiency is calculated according to the following equation:

8 $$IE=[({R}_{p}-{R}_{p0})/{R}_{p}]\cdot 100$$

where R_p0_ is the total polarization resistance of the copper electrode in SAR solution and R_p_ is the total polarization resistance of the in the presence of the inhibitor. The values of R_p_ are calculated following the equation $\,{R}_{p}={R}_{f}+{R}_{ct}$.

The calculated IE values are in agreement with the values obtained from potentiodynamic polarization and weight loss measurements.

### Weight loss measurements

In addition to the electrochemical measurements, the inhibitory ability of ibuprofen was also tested by using the weight loss method. The copper specimens were immersed for five days in the SAR and inhibited solutions, at room temperature. The effect of the different concentrations of ibuprofen on the corrosion rate is examined. From the results of the weight loss test, the values of the corrosion rate (*CR*) and inhibition efficiency (*IE*) were calculated using Eqs. (9) and (10) and the average values are summarized in Table 3:

9 $$CR=({W}_{0}-W)/A\cdot t$$

10 $$IE=[(CR-C{R}_{1})/CR]\cdot 100$$

Where *CR* and *CR*_1_ (g/m^2^h) are the corrosion rates of copper in synthetic acid rain in the absence and presence of inhibitor, respectively. *W*_0_ and *W* (g) are the weights of the copper samples before and after treatment in the appropriate solutions, respectively, while *A* (m^2^) is the surface area of the samples and *t* (h) is the immersion period.

**Table 3:** Weight loss parameters of copper oxidation in SAR solution and with the addition of different concentrations of ibuprofen.

Inhibitor concentration, M	CR, g/m^2^h	IE, %
/	0.0941	/
5·10^−4^	0.0363	61.4
1·10^−3^	0.0085	90.9
5·10^−3^	0.0065	93.1
1·10^−2^	0.0032	96.5

By analyzing these parameters, it can be seen that the corrosion rate decreases as the concentration of inhibitor increases. Additionally, the inhibition efficiency increases with increased ibuprofen concentration which agrees with the results obtained by the electrochemical techniques. It is assumed that a higher degree of copper surface is covered with a protective layer as the concentration of inhibitor increases. This leads to a decrease in corrosion rate in the SAR solutions. Additionally, the highest inhibition efficiency is achieved in the presence of 1·10^−2^ M ibuprofen, which is consistent with the results obtained in the potentiodynamic polarization experiments.

### Adsorption isotherm

**Figure 6:**
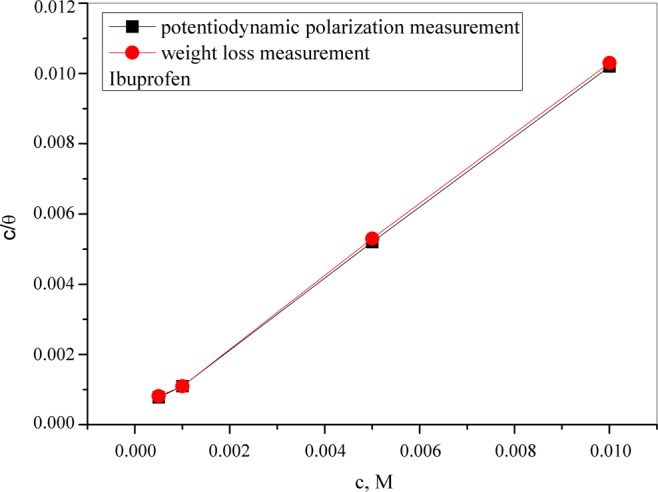
Adsorption isotherm of ibuprofen on the copper surface.

To obtain information about the type and degree of interaction between the copper surface and inhibitor molecules, adsorption isotherm studies are necessary. In this study, the obtained data are best fitted using Langmuir adsorption isotherm which is shown in Fig. 6:

11 $${C}_{inh}/\theta =1/K+{C}_{inh}$$

The straight line of C_inh_/θ vs. C_inh_ as well as the values of the regression coefficient (R^2^) and the slope (Table 4) confirms the adsorption of the ibuprofen molecules fits a Langmuir isotherm. This isotherm shows that the adsorbed molecule occupies only one active site on the electrode surface^88^. The Gibbs free energy of adsorption is calculated using the following equation:

12 $$-\Delta {G}_{ads}^{0}=R\cdot T\cdot \,\mathrm{ln}(55.5\cdot {K}_{ads})$$

Where R stands for universal gas constant, T is the thermodynamic temperature, 55.5 stands for molar concentration of water and K_ads_ represents the equilibrium constant of adsorption. In general, a high value of K_ads_ is associated with high adsorption efficiency indicating that ibuprofen under experimental conditions can be adsorbed. Thus, this is in good agreement with results obtained by electrochemical and weight loss measurements. The calculated value of the Gibbs free energy implies strong and spontaneous adsorption of ibuprofen molecules on the copper surface in the synthetic acid rain solutions.

**Table 4:** Langmuir adsorption parameters for ibuprofen on copper in SAR solution.

Technique	slope	R^2^	K_ads/des_∙10^4^, dm^3^/mol	−ΔG_ads_, kJ/mol
Potentiodynamic polarization	1.00	0.9996	1.93	31
Weight loss	1.01	0.9994	2.13	31

Considering the pH value of the tested SAR solution (pH 2.42) and the pKa value for ibuprofen (4.91)^89^, it is assumed that this compound is in a protonated form during the tests. The mechanism of inhibitor action could be due to adsorption of anionic species presented in SAR on the copper surface which further facilitates the adsorption of the protonated inhibitor.

### Surface characterization by scanning electron microscopy with energy dispersive spectroscopy

**Figure 7:**
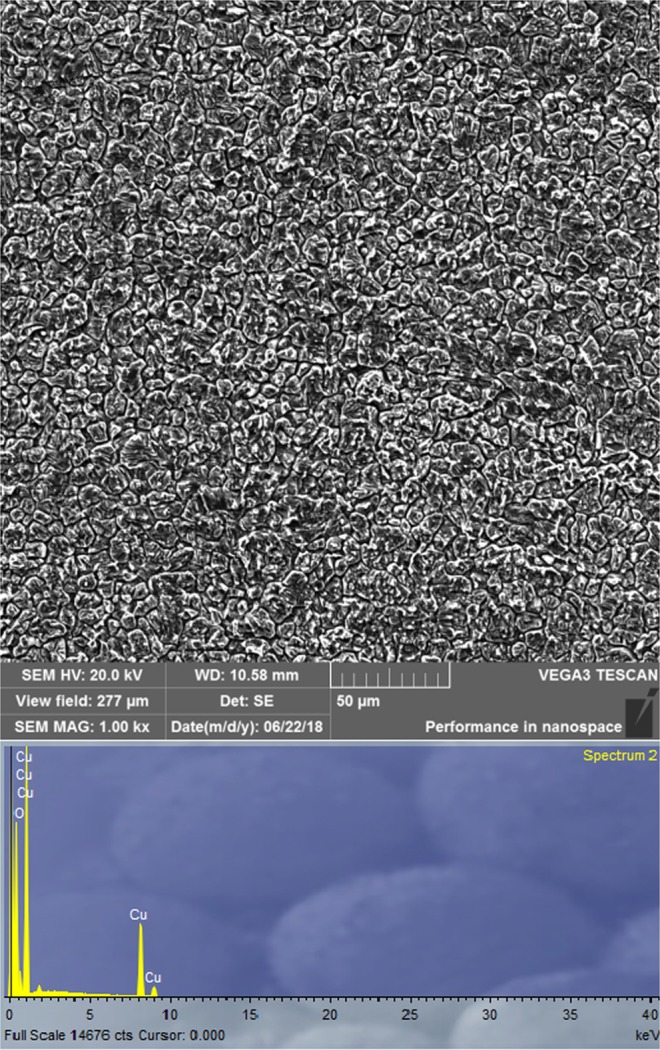
SEM images and EDS spectrum of the copper surface obtained after five days of immersion in an SAR solution.

**Figure 8:**
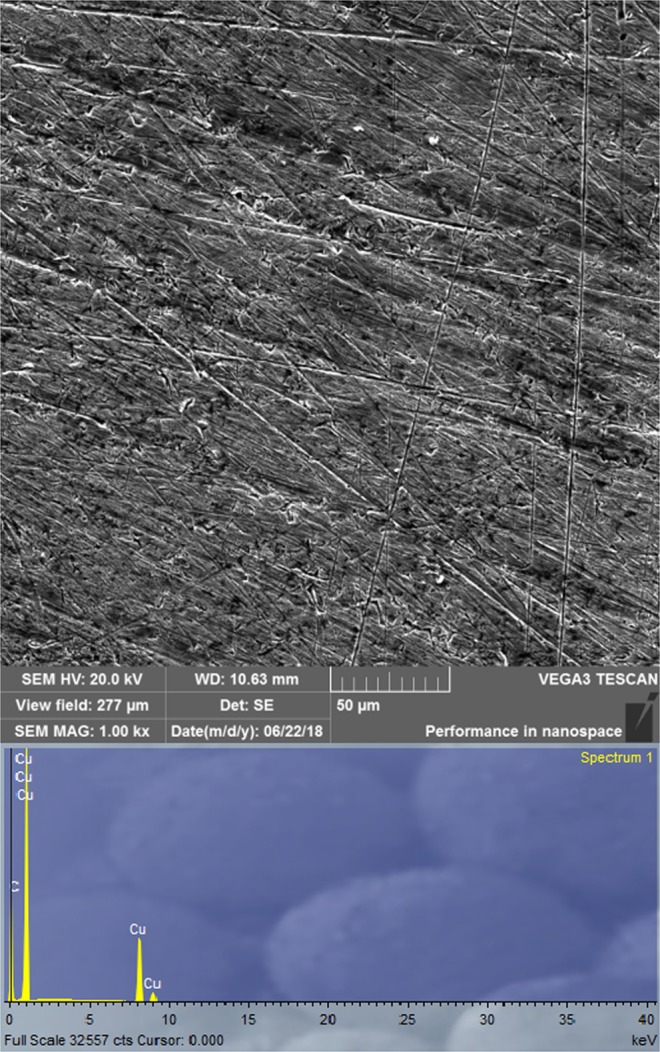
SEM images and EDS spectrum of the copper surface obtained after five days of immersion in an SAR solution with the addition of 1∙10^−2^ M ibuprofen.

The surface characterization of copper coupons treated in synthetic acid rain in the absence and presence of the highest concentration of ibuprofen is carried out by scanning electron microscopy with energy dispersive spectroscopy (SEM – EDS). The coupons were immersed for five days at room temperature in different solutions, and the obtained SEM micrographs are shown in Figs 7 and 8. By analyzing these figures it is seen that the copper surface is smoother in the presence of the inhibitor as opposed to the pits and cracks obtained in the inhibitor-free solution. This can be a result of the formation of a compact layer of ibuprofen on the metal surface.

According to the EDS results (Fig. 7) it is assumed that copper corrosion products are formed on the sample surface when inhibitor is not added to the SAR which agrees with the previously proposed corrosion mechanism. The copper coupon treated in the solution containing ibuprofen is also subjected to EDS analysis (Fig. 8). The absence of O atomic peak and also the presence of C atomic peak which is derived from inhibitor molecule leads to the conclusion that formed film hinders the formation of corrosion products. Hence, the presence of ibuprofen diminishes the corrosion rate of copper in SAR solution which is consistent with the experimental results. According to the EDS analysis (Figs 7 and 8) and CV curves (Fig. 3) obtained in inhibited solutions, it can be assumed that inhibitor molecules form complex with cuprous ions, thus formed complex is adsorbed on the copper surface and leads to decreasing the corrosion rate. Similar results are observed by Quartarone *et al*.^90^ and Tan *et al*.^91^.

### Quantum chemical calculations

**Figure 9:**
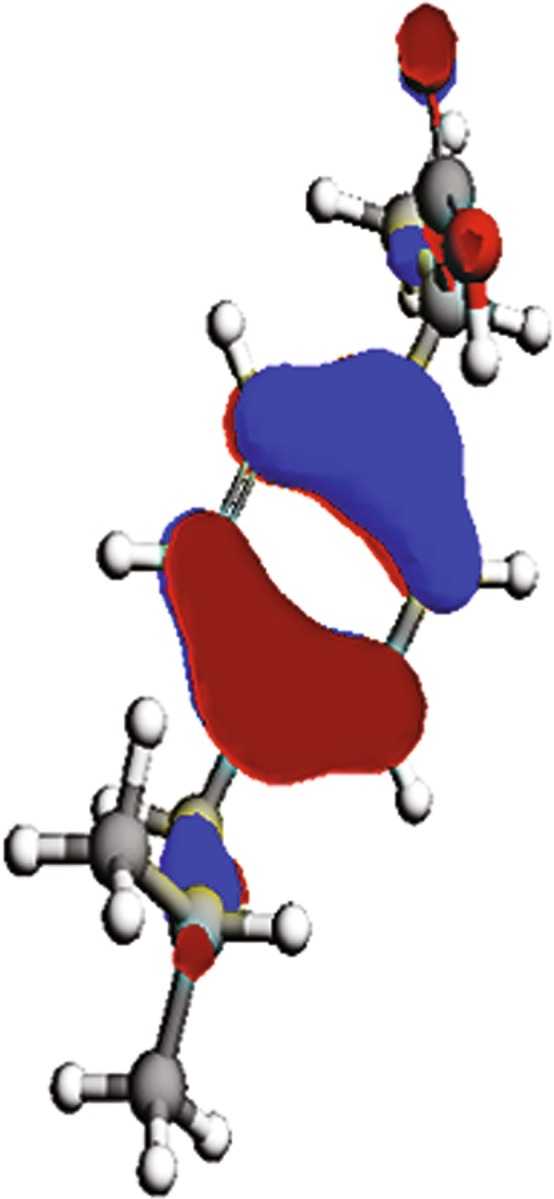
Distribution of the HOMO of ibuprofen.

**Figure 10:**
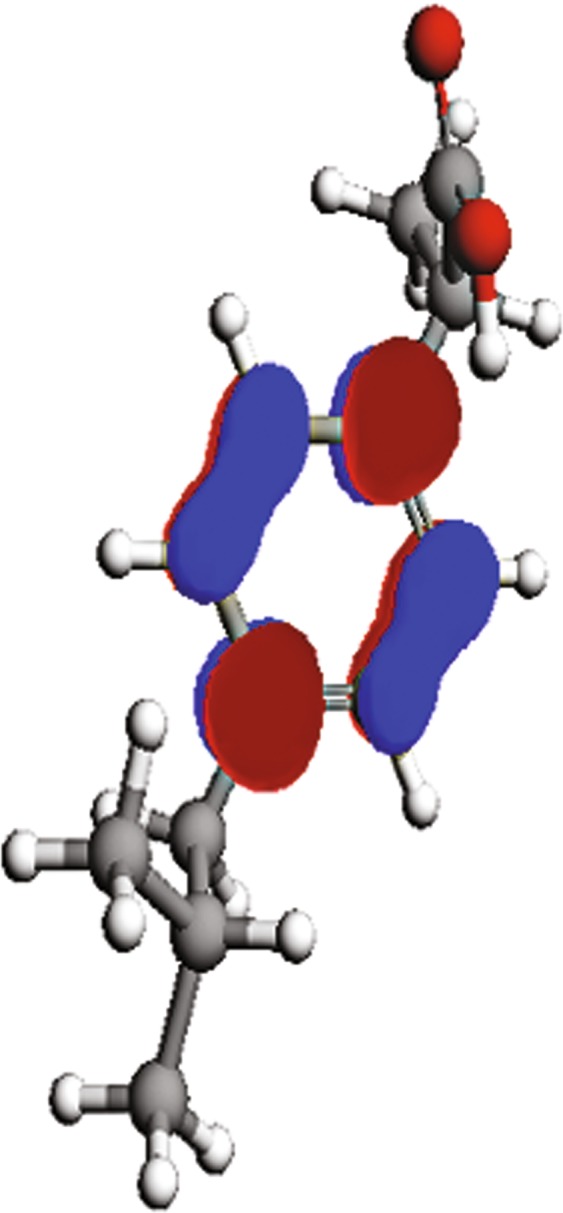
Distribution of the LUMO of ibuprofen.

To determine the relationship between the inhibition efficiency of the ibuprofen and its molecular structure, quantum chemical calculations have been performed. The molecular structure of ibuprofen has been geometrically optimized using DFT calculations performed with method using ArgusLab 4.0 software^92^ and that the following parameters have been calculated: the energy of the highest occupied molecular orbital (E_HOMO_), the energy of the lowest unoccupied molecular orbital (E_LUMO_), the energy gap barrier (ΔE) and the dipole moment (μ). Furthermore, ionization potential (I), electron affinity (A), electronegativity (χ), global hardness (η) and number of transferred electrons (ΔN) are calculated according to Eqs. (13) – (17). All the mentioned parameters are presented in Table 5. The spatial distributions of the HOMO (highest occupied molecular orbital) and the LUMO (lowest unoccupied molecular orbital) of ibuprofen are illustrated in Figs 9 and 10. The lower value of ΔE is associated with the higher affinity of the inhibitor molecules to be adsorbed on the metal surface^93^. According to this parameter, it is assumed that ibuprofen has high tendency to be adsorbed on the copper surface, which is consistent with the inhibition efficiency obtained in the experimental measurements. The lower electronegativity of ibuprofen also confirms high inhibition efficiency^94^.

13 $$I=-{E}_{HOMO}$$

14 $$A=-{E}_{LUMO}$$

15 $$\chi =(I+A)/2$$

16 $$\eta =(I-A)/2$$

17 $$\Delta N=({\chi }_{Cu}-{\chi }_{inh})/[2\cdot ({\eta }_{Cu}+{\eta }_{inh})]$$

where χ_Cu_ and χ_inh_ are the absolute electronegativity of copper (4.48 eV/mol) and the inhibitor molecule respectively, and η_Cu_ and η_inh_ are the absolute hardness of copper (0 eV/mol) and the ibuprofen molecule^95^. The higher value of the dipole moment of ibuprofen (4.29 D) than water (1.85 D) could be associated with a higher tendency of the ibuprofen to interact with the copper surface^94^. Due to the high value of dipole moment of ibuprofen, a high IE of this compound is expected^96^, which agrees with the results obtained by the electrochemical and weight loss measurements. On the bases of the quantum chemical parameters, the ibuprofen molecules have the ability to be adsorbed on the copper surface by replacing previously adsorbed water molecules^94^.

**Table 5:** Quantum chemical parameters.

Parameter	E_HOMO_, eV	E_LUMO_, eV	I, eV	A, eV	χ	η	ΔE, ev	μ, D	ΔN
Value	−9.62	−0.31	9.62	0.31	4.97	4.65	9.31	4.29	−0.05

## Conclusion

Expired ibuprofen has the ability to protect copper from corrosion in synthetic acid rain solution. According to results obtained by electrochemical and weight loss measurements, the inhibition efficiency raises with increase in ibuprofen concentration. Potentiodynamic polarization results classify ibuprofen as a mixed-type corrosion inhibitor. SEM and EDS analysis of copper coupons treated in SAR containing ibuprofen revealed the formation of a protective layer on the metal surface that reduced copper dissolution. The protective layer is formed by the adsorption of ibuprofen molecules on the copper surface according to a Langmuir adsorption isotherm. Quantum chemical parameters agree with the results obtained experimentally.

## Data Availability

In accordance with the institution’s policy, data are not available.
